# Microbial Musings – September 2020

**DOI:** 10.1099/mic.0.000978

**Published:** 2020-09-30

**Authors:** Gavin H. Thomas

**Affiliations:** ^1^​ Department of Biology, University of York, York, UK

As the British summer quickly changes into autumn, we have been making on some changes with the journal that should be worked through by the end of the year, as we start planning a series of events for our 75th Anniversary in 2022. The first of these to announce is the appointment of Andrew Preston (@apreston243) as our new Reviews Editor, who joins the team of senior editors at the journal. Andrew’s research broadly sits around bacterial pathogenesis, with particular interests in *Bordetella* sp. and their virulence factors, metabolism and evolution, and he is based at the Milner Centre for Evolution at the University of Bath, UK. Please send your ideas for full review articles or Insight Reviews to Andrew (A.Preston@bath.ac.uk), and early-career researchers, especially writing-up grad students, think about how your thesis introduction could be adapted for something timely for the journal.

The first paper highlighted from this issue also brings me right back to my time at grad school at the University of Birmingham, UK, in the mid-1990s, and reflections on how the best laid plans often need rapid adaptation! My PhD with Jeff Cole was predicated on the hypothesis that an *Escherichia coli* enzyme, the periplasmic nitrate reductase, Nap, was important for how *E. coli* is able to reduce the radionuclide technetium VII (soluble) to technician IV (insoluble), which was of great interest at that time to my industrial sponsors, British Nuclear Fuels. In my first presentation of the project to my research floor, an astute Nigel Savery (@nigelsavery), then a postdoc with Steve Busby FRS in the adjacent lab, asked me innocently enough what I would do if Nap was not involved in this process. I had not considered this possibility, of course, as it was written in the title of my PhD project and so must be true, and I was flummoxed! This motivated me to read deeply around anaerobic respiration in *E. coli *, which proved extremely valuable as Nigel was dead right and Nap was not involved in this process at all. Working with another young postdoc, Jon Lloyd (@profjrlloyd), and his boss, Lynne Macaskie, we figured out that instead it was hydrogenase 3 (Hyd-3) as part of the formate hydrogenlyase (FHL) complex that was the technetium reductase [1]. *E. coli* has multiple hydrogenases, which can catalyze either the oxidation or production of H_2_ and, as such, have potential applications in biohydrogen production. While I was working on this project the *E. coli* genome was sequenced [2], revealing an new operon, *hyf*, encoding a potential fourth system [3]. In the paper in this issue from the group of Frank Sargent (@Prof_Sarge), with Tracy Palmer (@proftracypalmer) and Sarah Coulthurst (@SarahCoulthurs1), the authors continue to investigate the still elusive function of hydrogenase 4 (Hyd-4) in *E. coli* [4]. Despite many people trying to find evidence for the function of Hyd-4, which has been proposed to form a second FHL, FHL-2, no clear demonstration of function has been demonstrated. Here the authors use a synthetic biology approach to create versions of the *hyf* operon on the chromosome with various strong promoters and the promoter from the *hyc* operon encoding hydrogenase-3, but see no evidence for H_2_ production by Hyd-4. Clearly, expression is not the bottleneck to function and they then switched to study a known post-translational regulatory step in the biosynthesis of the hydrogenase large subunit, where a protease, HycI, in the case of Hyd-3, is needed to complete maturation of the complex metal cofactor-containing enzyme. The *hyf* operon lacks an orthologue of HycI and, interestingly, in the related bacterium *Pectobacterium atrosepticum* the *hyf* cluster does contains a HycI homologue, HyfK. From doing various elegant complementation experiments between *E. coli* and *P. atrosepticum *, their data suggest that *E. coli* HycI should allow maturation of the Hyd-4, but, despite this, they know that no activity of FHL-2 can be detected, so the mystery continues around when and why this system is used.

Recounting my interactions with the Busby lab while at Birmingham also links me to another paper in this issue. In our joint lab meetings or over coffee we often heard about papers from the group of Akira Ishihama from Hosei University, Japan, who had usually done something novel and impressive with *E. coli* transcription factors (TFs). Ishihama was the first to try and study ‘all’ TFs from *E. coli *, rather than one or two that were usually studied by groups around the world at that time. Over the years his group have developed and applied a range of technologies to elucidate the function of many TFs in *E. coli* [5], and through their work, and that of many other groups around the world, there are now 145 *E. coli* TFs with known function – still leaving another 53 with unknown physiological roles [6]. In this paper from the group of Hiroshi Ogasawara at Shinshi University, Japan, working with Ishihama’s group, they exploit one of Ishihama’s amazing resources, which is a library of 198 purified transcription factors from *E. coli* [6, 7]. They use these proteins in a method they call the ‘TF-to-promoter’ approach, where each protein is used individually in a gel-shift assay with the promoter sequence being tested. Here they apply this method to an important promoter in *E. coli *, that for the *csgD* gene, which encodes the master regulator of biofilm formation. Expression of *csgD* is under complex control with already 14 known TFs linked to this promoter and remarkably 48 of the TFs from their library bind strongly to the *csgD* promoter, including 35 known and 13 uncharacterized TFs. To learn more about the uncharacterized TFs, they study seven of them in more detail using complementary approach to confirm their *in vitro* results. They find that they can obtain strong evidence for two of these uncharacterized TFs, YiaJ (PlaR) and YhjC (RcdB), regulating the *csgD* promoter. For RcdB this is the first evidence for any promoter it regulates, while PlaR is a TF that they think from a related study is involved in regulating operons involved in the utilization of plant-derived nutrients, although this still needs further study [8]. The study reminds us that we still have plenty of biology to learn from this model organism, over 20 years after its genome was sequenced.

The next two papers featured from this month’s issue both relate to cell division in Gram-positive bacteria. The first keeps us at the University of Birmingham, with a paper from Hayleah Pickford (@ModMedMicro) from her work in Apoorva Bhatt’s group at the IMI (@IMIBirmingham), in collaboration with Gabriella Keleman at the University of East Anglia, UK. In this work the authors are looking at the product of the *MSMEG_2416* gene from *Mycobacterium smegmatis *, which is a coiled-coil protein like the homologous DivIVA protein known to be involved in cell division [9]. Filamentous bacteria such as *Mycobacterium* and its actinomycete cousin *Streptomyces coelicolor* exhibit polar growth, where the new cell develops at the cell pole rather than the mid-point. DivIVA forms a component of the tip-organizing centre (TIPOC), which is required for this polarized growth. In this work they were able to construct a strain of *M. smegmatis* with a disruption in the *MSMEG_2416* gene, which contrasts with two other studies where this gene had been concluded to be essential [10, 11]. The mutant does have altered colony morphology, which is restored by complementation, but the altered morphology is not due to changes in lipid composition in the mutant. The cells are also elongated in the mutant and have altered septation patterns. The authors conclude that *MSMEG_2416* is important for mycobacterial growth and reflect on how they were able to create a conditional mutant, whereas the other groups were not.

Our second ‘div’ paper is on another Gram-positive bacterium, *Bacillus subtilis *, but one that divides at mid-cell, unlike the more unusual actinobacteria described above. The divisome apparatus that mediates cell division contains many interacting proteins that coordinate the synthesis of new peptidoglycan and correct protein–protein interactions within the divisome are critical for its function. In this paper from the group of Dirk-Jan Scheffers (@DirkScheffers) the authors examine the function of PASTA domains between some of the divisome components and discover an important function in holding together the DivIB regulator of division with the essential penicillin-binding protein 2b (PBP2b), which has PASTA domains at its C-terminus [12]. They demonstrate this nicely using a bacterial two-hybrid system and then by co-immunoprecipitation, and conclude the PASTA domains are important scaffolding components within the divisome, but that they are not essential.

One thing I always found interesting during the short time I worked on *Pseudomonas aeruginosa* as a postdoc, was that glucose was not its preferred source of carbon, in fact given both glucose and the TCA-cycle intermediate succinate, it exhibits a diauxie but uses the succinate first [13, 14], in stark contrast to *E. coli *, which uses glucose first. One of the organic acids that it can grow on is the C5-dicarboxylate α-ketoglutarate (α-KG), which is transported into the cell by a proton-coupled symporter, the expression of which is regulated by a two-component regulatory system (TCS) called MifS/MifR, with MifS being the sensory subunit [15]. The protein is from the same family as the DctB histidine kinases that are known to sense related C4-dicarboxylates such as fumarate and succinate [16]. The sensor has two transmembrane domains, between which is a structured periplasmic domain that binds directly the molecule to be sensed, which results in the transduction of this signal through the membrane to the transmitter domain in the cytoplasm. Zaara Sarwar from The College of New Jersey, USA, working with the group of Christopher Nomura at the State University of New York, Syracuse, USA, has been looking at the function of MifS and has found that it is highly specific for α-KG and only recognizes the related C5-dicarboxylate glutarate [17]. There are structures of similar sensing domains such as the *Vibrio cholerae* DctB protein, which adopts α/β PDC fold [18], and the authors modelled the MifS sensing domain on these. Using a combination of mutagenesis and a *lacZ* reporter to measure activity of MifS, they were able to describe the likely α-KG binding pocket in the protein. They nicely demonstrate that their model is correct by changing a residue that they predict discriminates between α-KG and succinate and find that the resulting protein will now respond to succinate. The inability to bind any other dicarboxylates makes MifS distinct from DctB and expands the functional range of this class of sensors, with this being the first known direct sensor of α-KG characterized to date.

Finally, check out this month’s Editor’s Choice, selected by Senior Editor Gail Preston, concerning the function of the AlgP regulon in *P. aeruginosa *, from Ashley Cross (@PresidentCross) in the group of Joanna Goldberg at Emory University School of Medicine, USA with ex-Senior Editor Marvin Whiteley (@whiteleylab) [19]. And look out for our adverts for Editors, Senior Editors and our Deputy Editor in Chief – come and join us and help #publishingforthecommunity as we take the journal forward to its big celebration in 2022.
